# An Account of the Successful Employment of Catgut in a Case of Fistula in Perinæo

**Published:** 1788

**Authors:** G. Wilkinson

**Affiliations:** Surgeon at Sunderland, and Member of the Royal College of Surgeons of Edinburgh


### III. *An Acount of the ſucceſsful Employment of Catgut in a Caſe of Fiſtula in Perinæo. Communicated in a Letter to Dr.* Simmons, *F. R. S. by Mr.* G. Wilkinſon, *Surgeon at Sunderland, and Member of the Royal College of Surgeons of Edinburgh.*

THE ſubject of this caſe was a ſea-faring man, aged about forty-ſeven years. In the year 1786, eight months before I ſaw him, he had contracted a gonorrhœa, and in the courſe courſe of this complaint, in conſequence, as he ſuppoſed, of expoſure to violent cold, inflammation had taken place in the perinæum, and the latter had ſwelled to an enormous ſize. On the fourth day a diſpoſition to gangrene began to take place; and on the eighth or ninth one half of the ſcrotum, and part of the perinæum, had ſloughed off.

The parts ſoon acquired a diſpoſition to heal; but from this period the urine began to flow through an opening in the perinæum, and very little of it paſſed through the penis.

At the time he applied to me I found two apertures with callous edges; one of them in the poſterior part of the ſcrotum, which it had contracted into ſeveral folds or rugæ; the other near to the anus, in the membranous portion or bulb of the urethra, the whole of which was much indurated; and it was from this part principally that the urine eſcaped.

I could not learn that any attempt had been made to dilate or open the natural paſſage for the flow of urine; but from the moment the patient came under my care I loſt no time in attempting to accompliſh this. I accordingly tried to introduce bougies of different ſizes, and ſome of them were retained in the urethra, againſt

 the the obſtruction, for about two hours in the day, for ſeveral days together. Some of theſe bougies had a ſmall wire in their center, and I even attempted the introduction of a fine ſmall leaden catheter; but I did not perceive that any progreſs was made in removing the obſtruction.

At this period, happening to mention the caſe to an ingenious ſurgeon of this place, he adviſed me to make trial of the catgut; adding, that he had ſeen it ſucceed in removing a ſtricture of the urethra of many years ſtanding, after every other effort had failed.

I immediately determined to have recourſe to this method, and, after ſeveral trials made with different catguts, (the ends of which I had previouſly ſmoothed with a file and oiled) I at length ſucceeded ſo far as to introduce, beyond the obſtruction, the third ſtring of a violin. This remained in the urethra about two hours; and the paſſage being dilated by the expanſion of the ſtring, I paſſed in a longer one, which remained in nearly the whole night; and the day following I was enabled to introduce with eaſe a ſmall bougie, which paſſed as far as the bladder.

The obſtacle being thus got over, the bougies were increaſed in ſize, by degrees, to that of a large large quill. The hollow bougie was likewife tried, but found not to anfwer fo well as the other. The patient's belly was kept open; and the irritation occafioned by the bougies (for he frequently retained them the whole night, and the greateft part of the day) was relieved by opiates. Mercurial ointment was rubbed in about the perinæum; and fmall dofes of calomel were given internally. Under this mode of treatment the urine continued to pafs freely through the urethra, and the cure was compleated in about fix weeks.

This method of removing obftructions in the urethra by means of catgut, though recommended by Le Dran in his operations and confultations in furgery, and mentioned by Sharp in his critical inquiry into the prefent ftate of furgery, is but little noticed by later writers, and, according to the information I have been able to collect, is not generally known.

Thefe confiderations have induced me, Sir, to communicate to you this inftance of the fuccefs attending this mode of treatment, to be inferted, if you think proper, in the London Medical Journal.

Since this cafe occurred, I have had an opportunity of trying, with fuccefs, the effects of the catgut in two other inftances of ftricture of the urethra from venereal caufes.

*Sunderland,*

*September 15th, 1788.*

